# COVID-19 pandemic response and Field Epidemiology and Laboratory Training Programs in ECOWAS

**DOI:** 10.11604/pamj.2024.48.49.42504

**Published:** 2024-06-07

**Authors:** Virgil Kuassi Lokossou, Azuka Stephen Adeke, Chukwuma David Umeokonkwo, Laurent Comlan Mariame Bonkano, Aishat Bukola Usman, Lionel Sogbossi, Appolinaire Kima, Patrick Nguku, Simon Antara, Issiaka Sombie, Felix Agbla, Melchior Joel Codjovi Aissi

**Affiliations:** 1 ECOWAS Regional Center for Surveillance and Disease Control, Abuja, Nigeria,; 2 Department of Community Medicine, Alex Ekwueme Federal University Teaching Hospital, Abakaliki, Nigeria,; 3 African Field Epidemiology Network, Kampala, Uganda,; 4 African Field Epidemiology Network, Abuja, Nigeria,; 5 West African Health Organization, Bobo-Dioulasso, Burkina Faso

**Keywords:** COVID-19 pandemic, field epidemiology and laboratory training programme, lessons learnt, ECOWAS

## Abstract

The COVID-19 pandemic has been persistent with a huge demand for human health resources which is a vital component of its preparedness and response. Globally, the public health workforce through field epidemiology and laboratory training programme (FELTP) has been instrumental to global health security. We determined the status of FELTP in the region and its contributions to the COVID-19 pandemic response in the ECOWAS region. We conducted a desk review, shared a questionnaire among member states and organized a two-day online regional consultative meeting on field epidemiology training on 30^th^-31^st^ March 2022 during which there were presentations, group discussions and deliberations on the status and contribution of FETP during the COVID-19 pandemic. Data collected were analyzed in themes. All countries in the ECOWAS region had established at least one tier of FELTP, 11 (73.3%) had established two tiers of FELTP and only 3 (20.0%) had established all three tiers of the program. Despite the pandemic, the cumulative number of graduates increased from 2996 to 4271 frontline, 41 to 380 intermediate, and 409 to 802 for advanced FELTP between 2019 and 2022. However, the progress has been disproportionate across countries. The key activities supported through FELTP graduates included pandemic response coordination, surveillance, data collection/management, laboratory support, case management, risk communication, infection prevention and control, COVID-19 vaccination, and research. Despite improvements in the FELTP in the Economic Community of West African States (ECOWAS) region, there is a need for continuous stakeholder engagement for its implementation, resource mobilization for sustainability, and leveraging critical partnerships.

## Introduction

The COVID-19 pandemic unveiled the gaps in global health preparedness, surveillance, response, and collaboration in general. It also reveals the internal struggles between focusing on national health security versus working together to ensure global health security, isolation versus collaboration among countries, and focusing on preventive versus curative health. The pandemic strengthened the need to ensure each country can quickly identify health threats, contain them at source and prevent them from spreading beyond the point of identification to other areas through a collaborative open and responsible partnership.

In the West African region, there were fears about the likely impact of the pandemic, given the weak health system and the impact of the 2005 Ebola virus disease outbreak. However, several factors have worked together to mitigate the effect of the pandemic in the region. In 2019, the West African Health Organization (WAHO), as part of its effort to strengthen the public health workforce capacity development within the Economic Community of West African States (ECOWAS) region organized a regional consultative meeting with member states (including Mauritania) to share experiences, identify gaps, and discuss strategies for strengthening and sustaining Field Epidemiology and Laboratory Training Programs (FELTPs) [1]. WAHO has been promoting training in field epidemiology in the ECOWAS region through the Regional Center for Surveillance and Disease Control (RCSDC). The region has made some progress on public health manpower development.

Across the globe, public health workforce development through FELTP has been instrumental to the COVID-19 pandemic response [2]. The COVID-19 pandemic has revealed important progress and gaps in the role of FELTP in tackling major public health emergencies [3]. Among these are human capacity gaps, funding gaps, the ease of mobilization and deployment of trained manpower to the places where they are needed most, and the need to innovate on the way the training is being delivered.

Overall, the region has achieved only 23% of the minimum recommended target set by the 2005 International Health Regulation Joint External Evaluation indicators as of the end of 2018. However, a lot of disparities exist at the country and sub-national levels [1,4]. It is estimated that each district (or its equivalent) in the region requires 5-7 health workers with basic FELTP (frontline) capabilities to build the capacity of surveillance and response staff. The region with over 2,000 districts (or their equivalent) needs at least 12,000 frontline epidemiologists but had about 2996 such capacities as of August 2019 [1].

In this paper, we reviewed the experiences of ECOWAS member states in responding to the COVID-19 pandemic, leveraging on their available public health workforce, and exploring the lessons learnt, as well as the strategies for building the region's capacity against future public health emergencies.

**The West Africa health setting:** the West African region comprises 15 member states of the ECOWAS. These countries share cultural, geopolitical, and economic ties with a highly mobile cross-border movement, diverse sub-national communities, and weak health systems. These possibly contribute to the ease of disease spread within the region. Recent examples include the Ebola virus disease outbreak, the measles outbreak and the COVID-19 pandemic. Member states have been strengthening the regional capacity to prevent and mitigate these outbreaks when they occur through different engagements coordinated by WAHO. As part of the regional capacity development and health system strengthening RCSDC organised a regional review meeting in March 2022 to assess the progress of public health workforce development in the region and the contribution of field epidemiologists within ECOWAS in the COVID-19 pandemic response.

**Stakeholder engagement:** we conducted a desk review of the status of FELTPs in West Africa, using country-level program reports which included project reports (e.g. Regional Disease Surveillance Systems Enhancement project), institutional reports, and country progress reports. In addition, journal articles on FELTPs in the ECOWAS region were reviewed. Also, the number of required epidemiologists (intermediate and frontline levels) was determined for member states using the United Nations Department of Economic and Social Affairs [5].

Directors of FELTP and Directors of Public Health or any national official leading the FELTPs in member states received a questionnaire by email that sought feedback on the status of the FELTP and their role during the pandemic. The questionnaire excluded details of respondents, and confidentiality was maintained. RCSDC organized a 2-day online regional consultative meeting on FELTP on 30^th^-31^st^ March 2022. The objective of the meeting was to discuss strategies for strengthening the FELTP in the ECOWAS region and the contributions of FELTP to the COVID-19 pandemic response in West Africa.

Meeting participants included senior officials of Ministries of Health, representatives of national public health institutions, African Field Epidemiology Network (AFENET) representatives, resident advisors, FELTP program supervisors, academic institutions, FELTP mentors, and representatives of technical and financial partners such as World Health Organization (WHO), Africa Centers for Disease Control and Prevention (Africa CDC), US Centers for Disease Control and Prevention (US CDC), Food and Agriculture Organization (FAO), and Regional Animal Health Center. The meeting methodology included plenary sessions and group discussions moderated by regional experts and highlighting progress made in implementing FELTP. Member states made presentations during plenary sessions to share the status of their programs. Their presentations focused on the in-country status of FELTP during the COVID-19 pandemic, FELTP training efforts, key activities of FELTP graduates and trainees, best practices/innovations and lessons learnt from the COVID-19 response. Participants also engaged in group discussions to chart a way forward for FELTP in West Africa. Several recommendations were made to strengthen field epidemiology in the region. All the quantitative data were summarized with frequencies and proportions. Detailed content analysis was done to generate themes for the qualitative data.

**Figure 1:**
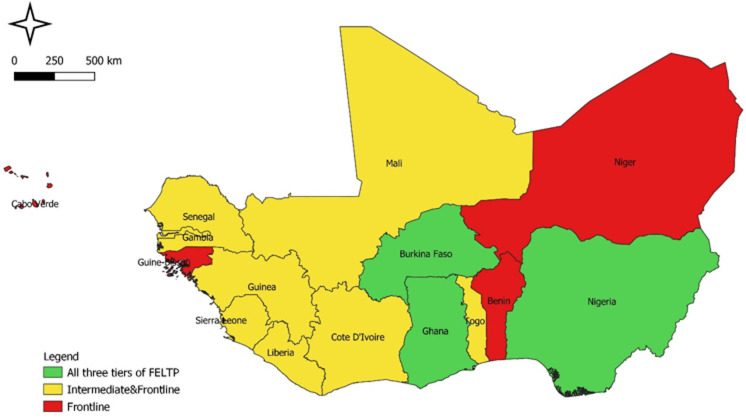
status of Field Epidemiology and Laboratory Training Program implementation by level of training, West Africa, March 2022

**Status of Field Epidemiology and Laboratory Training Program in ECOWAS region during COVID-19 pandemic response:** at the end of March 2022, all 15 ECOWAS countries had established FELTP or applied epidemiology training programs. Cabo Verde and Niger, which had not started FELTP in 2019, had established frontline FELTP. Nine countries had two tiers of FELTP and included Cote d'Ivoire, The Gambia, Ghana, Guinea, Liberia, Mali, Senegal, Sierra Leone, and Togo. Only Burkina Faso, Ghana, and Nigeria had the three levels of the program (Figure 1).

ECOWAS region had 4,271 frontline, 380 intermediate, and 802 advanced FELTP graduates as of March 2022. However, many countries have not met the minimum target on the number of epidemiologists needed, countries like Nigeria, Cote d'Ivoire, Niger, Mali, Burkina Faso, Benin, Cabo Verde and Mali are still below 50% of the minimum target of epidemiologists expected. Few countries like Liberia, Ghana, Sierra Leone and Gambia have met their minimum required number of epidemiologists though the distribution of these critical human resources in the countries and retaining them within the public health system of the country remains a challenge (Table 1). During the pandemic (2020/2021), all the countries in the region implemented frontline FELTP except Benin and Nigeria, and intermediate FELTP except Benin, Cabo Verde, Guinea-Bissau and Niger (Table 2). Also, all the countries had trainees undergoing the advanced FELTP training except Cabo Verde.

**Table 1:** distribution of FELTP graduates in countries by level and percentage of target reached, West Africa, March 2022

Country	Frontline	Intermediate = a	Advanced = b	Total Epidemiologists available = a+b	Estimated Population in millions (March 2022)	Minimum Required Number of Epidemiologists for Country	Percentage Coverage %	Epidemiologists still needed	Epidemiologists in training in 2022
	FELTP Graduates								
Benin	100	0	9	9	12.9	64	14.1	55	0
Burkina Faso	306	24	13	37	21.5	107	34.6	70	16
Cabo Verde	46	0	0	0	0.5	3	0	3	0
Cote d'Ivoire	388	14	12	26	26.5	132	19.7	106	37
Gambia	208	15	13	28	2.4	12	233.3	-16	5
Ghana	486	69	154	223	31	155	143.9	-68	77
Guinea	213	35	11	46	13.6	68	67.6	22	26
Guinea-Bissau	198	0	7	7	2	10	70	3	0
Liberia	267	80	28	108	5.3	26	415.4	-82	35
Mali	165	15	12	27	22.8	114	23.7	87	16
Niger	29	0	18	18	22.3	112	16.1	94	12
Nigeria	1116	14	495	509	214.6	1073	47.4	564	0
Senegal	263	34	10	44	16.2	81	54.3	37	18
Sierra Leone	224	54	11	65	8.6	43	151.2	-22	17
Togo	262	26	9	35	8.2	41	85.4	6	24
Total	4271	380	802	1182	408.4	2042	57.9	1045	283

**Table 2:** FELTP training effort during the COVID-19 pandemic, West Africa, March 2022

Country	Frontline FELTP			Intermediate FELTP			Advanced FELTP		
	December 2019 COVID-19	Available March 2022	September 2021 COVID-19	December 2019 COVID-19	Available March 2022	September 2021 COVID-19	December 2019 COVID-19	Available March 2022	September 2021 COVID-19
**Benin**	100	100	0	0	0	0	9	9	0
**Burkina Faso**	266	306	40	0	24	24	5	9	4
**Cabo Verde**	0	46	46	0	0	0	0	0	0
**Cote d'Ivoire**	335	388	53	0	14	14	4	8	4
**Gambia**	144	208	64	0	15	15	1	13	12
**Ghana**	359	486	127	0	69	69	88	154	66
**Guinea**	114	213	99	0	35	35	4	11	7
**Guinea-Bissau**	180	198	18	0	17	17	2	5	3
**Liberia**	189	267	78	30	80	50	2	28	26
**Mali**	112	165	53	0	15	15	7	19	12
**Niger**	0	29	29	0	0	0	6	18	12
**Nigeria**	1116	1116	0	0	14	14	277	495	218
**Senegal**	181	263	82	0	21	21	4	7	3
**Sierra Leone**	103	224	121	11	54	43	1	11	10
**Togo**	116	262	146	0	13	13	7	14	7

**Figure 2:**
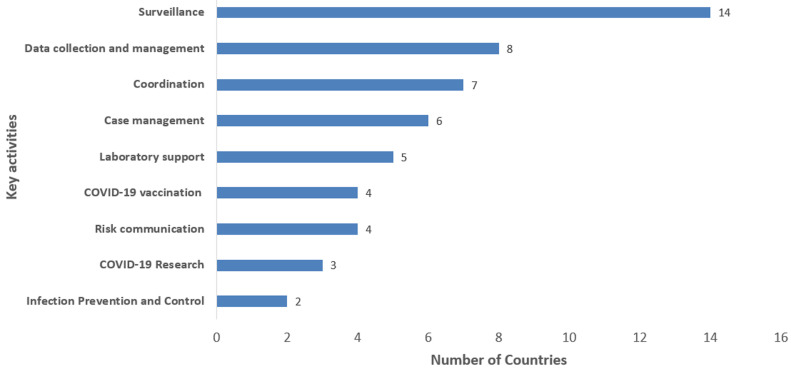
key activities conducted by FELTP graduates and trainees during COVID-19 pandemic response, West Africa, March 2022

**Contributions of FELTP graduates and trainees during the COVID-19 pandemic:** in all ECOWAS member states, FELTP graduates and trainees were greatly involved in the COVID-19 pandemic response in their countries (Figure 2). Key activities supported through the FELTP with its graduates and trainees were coordination; surveillance (e.g. alert/case investigation, contact tracing); data collection and management; laboratory support (e.g. sample collection and testing); case management; risk communication; infection prevention and control; COVID-19 vaccination; and research. For example, Benin engaged six frontline FELTP consultants to provide technical support on different aspects of the pandemic response. In Cote d'Ivoire, residents and graduates participated in multiple COVID-19 vaccination campaigns. Countries like Liberia conducted COVID-19-related studies such as vaccine hesitancy study. For Nigeria, many graduates and trainees occupied leadership positions during the COVID-19 pandemic and were deployed into rapid response teams, teams in points of entry, and surveillance. In Burkina Faso, FETP graduates and trainees were deployed as rapid response team members. In Cabo Verde, the FETPs participated in the training of central and local surveillance teams. They revised the weekly bulletins on the epidemiological situation of COVID-19 and produced an immunization bulletin. In Ghana, the FELTPs graduates and residents contributed to intensifying the national response. They developed national protection protocols and conducted PoE screening, laboratory testing, district support and community surveillance.

**Best practices/innovations during the COVID-19 pandemic response:** across the different FELTPs in West Africa, different best practices were reported during the COVID-19 pandemic response. For most countries, FELTP graduates and trainees were easily mobilized and deployed to join rapid response teams and they were available to be engaged. In Burkina Faso, the program reported smooth collaboration among the different levels of FELTP officers with weekly reporting of their activities. In Nigeria, FELTPs were the pillar leads of the rapid activation of the National Emergency Operations Center for the response [6]. Online meetings were widely conducted in countries due to the risk of transmission of the disease. The pandemic also led to other online-related engagements such as online mentorships, the use of social media for pandemic response activities such as risk communication, adaptation of FELTP training curriculum to involve virtual sessions during lockdown periods. For example, in conducting the field epidemiology training, some countries like Liberia adapted their pre-admission course to an online format to comply with the COVID-19 restriction and public health advisory required during the pandemic [7]. As an innovation, Guinea FELTP highlighted the use of a single platform for entering COVID-19 data (vaccination, surveillance, etc.). They also mentioned the introduction of COVID-19 immunization card through the DHIS2 platform, Guinea uses the software K-Santé for contact tracing. During the COVID-19, countries like Nigeria included civil societies and local communities for the response. They activated the national EOC at the NCDC. A multi-sectoral COVID-19 Presidential Task Force was set up with decisive action to halt international travel and impose a time-bound lockdown in the heavily affected areas.

FELTP graduates and trainees were used to ensure the continuity of essential health services like immunization, and nutrition, and also integrated maternal and child health care at the community level. Furthermore, there were innovative approaches adopted in the response which included synchronization of COVID-19 data (e.g. surveillance, vaccination, etc.) in Guinea, and integration of COVID-19 surveillance and Acute Flaccid Paralysis (AFP) surveillance activities by FELTP personnel in Togo. They were also involved the various field research to assess the impact of the pandemic on health and make the necessary adjustments to response activities.

**Lessons learnt from the response of FELTP graduates and trainees during the COVID-19 pandemic:** the COVID-19 pandemic has shown more relevance of field epidemiologists to respond to outbreaks within the West African region. Lessons learnt for the success of the pandemic response are the strengthening of One Health multisectoral approach, political will at the national level and local commitments at the sub-national level. Other lessons learnt included enhancing the routine surveillance system through improved data quality and skills of surveillance officers, use of virtual training methods, strengthening research capacity of FELTP officers and capacity building for FELTP mentorship. Following the pandemic, there is need for continuous stakeholder engagement for FELTP implementation, continuous resource mobilization necessary for sustainability, and leveraging critical partnerships in the FELTP space.

Stakeholders made recommendations to strengthen field epidemiology and these include improvement of post-training follow-up of FELTP graduates, extending the annual consultation platform for field epidemiology training to other partners, and accelerating the networking process for FELTP officers in the West African region. Other key recommendations were the strengthening of FELTP institutionalization programs within countries, organizing regional strategic communication coaching courses for already trained FELTP beneficiaries, and engagement of technical and financial partners to increase FELTP budget to make up for the shortage of field epidemiologists in the ECOWAS region.

## Discussion

Our study reported that all the ECOWAS countries currently have at least one of the levels of FELTP ongoing within their country. This is an improvement from a study report in 2019 with findings of some countries in the West African region not having any level of the FELTP [1]. Globally and even in Africa, there is a severe shortage of field epidemiologists [8]. Hence, at the level of the West African region, efforts are being made to increase the field epidemiology workforce through the engagement of Ministries of Health, national public health institutions, field epidemiology academic institutions, and technical and financial partners (WHO, Africa CDC, US CDC, FAO, and AFENET).

This seems to be yielding some positive impact as seen from this study. An earlier assessment had reported that the number of frontline, intermediate, and advanced graduates were 2996, 41, and 409 respectively as of 2019 [1]. However, findings from this study have shown an increase in the number of graduates in 2022 to 4290 for the frontline, 380 for the intermediate, and 802 for the advanced level. It is worthy of note that this increase has been disproportionate across the countries. For example, while other countries have increased their frontline graduates since 2019, Nigeria and Benin did not record any new graduates from the frontline level of FELTP. For the intermediate level, while only two countries (Liberia and Sierra Leone) had intermediate graduates in 2019, others except Benin, Cabo Verde, Guinea Bissau, and Niger have commenced training and now have intermediate graduates. On the advanced level of FELTP, although only Burkina Faso, Ghana, and Nigeria have advanced-level training in-country, many other countries have increased their number of advanced-level graduates except Cabo Verde and Mali. The COVID-19 pandemic has been a good opportunity for investing and strengthening FELTP in the ECOWAS region. In addition to the support received from partners, member states have also allocated domestic funding to increasing cohorts of residents and improving the quality of training.

The differences observed in FELTP between the countries in the ECOWAS region may be due to various factors. These factors include local commitment, political will, government funding, and partners' support. Each ECOWAS country needs to improve its local commitment and political will towards FELTP to increase and strengthen its public health workforce. Also vital to the success of FELTP in countries is funding; this includes funding from both the government and partners like the US CDC. Other partners' support needed by countries include technical support and indigenous capacity building to achieve country ownership of the training programs. Our findings suggest that eight francophone countries have access to only one advanced-level program. It is vital to consolidate the above gains and strengthen field epidemiology capacity development across all the ECOWAS countries, with a particular focus on the francophone countries. There is an urgent need to establish an additional advanced program for the francophone member states.

For many ECOWAS countries such as Nigeria, Cote d'Ivoire, Mali, Niger, Burkina Faso, Benin, and Senegal, over 50 epidemiologists are still required with Nigeria needing as high as 564 more epidemiologists. Hence, there is a need for continuous stakeholder engagement for improved FELTP implementation, continuous resource mobilization for sustainability, and leveraging critical partnerships. Our study also revealed the urgent need for tailoring support to some countries in advanced FELTP programmes. In different parts of the world, the training programs are being evaluated to suit local demands [9].

Our study has shown that graduates and trainees of FELTP in the West African region have been greatly involved in the COVID-19 pandemic response with the different activities and pillars of the response. Across all six WHO regions, residents and graduates of FELTP have engaged in the pandemic response through rapid response teams, coordination, surveillance, case investigations, activities at points of entry, risk communication and community engagement [10]. Other engagements that involved FELTPs in lower percentages were national laboratories, infection prevention and control, case management, operational support and logistics. Some earlier reports from West Africa have related some of the activities of FELTP graduates and trainees in Ghana and Nigeria [9].

A major strength of this study is its inclusion of findings from all the countries in the ECOWAS region. These findings are key to addressing field epidemiology in every West African country. Despite this strength, a limitation of our study is that the reports presented by different countries were not verified by any team of experts within the region.

## Conclusion

For the FELTPs in the West African region, lots of lessons have been learnt from the pandemic response of FELTP graduates and trainees. The COVID-19 pandemic emphasizes the relevance of field epidemiologists for outbreak responses within the region. ECOWAS region has reported a significant increase in FELTP graduates during the COVID-19 pandemic response. Our findings underscore the value of FELTPs to build the field epidemiology workforce capacity that is so critical during responses to public health emergencies. Adequate field epidemiology workforce capacity takes time to develop and requires investment from national governments and donors as well as political commitment. There is also a need to develop a strategic plan for ECOWAS member states and partners in the region to address gaps in knowledge and skills in field epidemiology, in the context of national and regional disease surveillance systems and multiple health sectors. As a region, we will continue to engage relevant stakeholders which include governments and partners for improved FELTP implementation.
